# Case Report: Novel Mutation of F5 With Maternal Uniparental Disomy Causes Severe Congenital Factor V Deficiency

**DOI:** 10.3389/fped.2022.913050

**Published:** 2022-06-07

**Authors:** Lin Cheng, Ying Li, Wenjuan Zhou, Tao Bo

**Affiliations:** Division of Neonatology, Department of Pediatrics, The Third Xiangya Hospital, Central South University, Changsha, China

**Keywords:** congenital factor V deficiency, coagulation factor V, factor V gene, mutation, uniparental disomy

## Abstract

We summarized two cases of congenital factor V deficiency (FVD) associated with a novel F5 mutation, and analyzed the relationship of the clinical features and genetic characteristics in congenital FVD. Case 1 was a female newborn infant with remarkable bleeding who died of severe intracranial hemorrhage on day 42 after birth. She had significant prolongation of prothrombin time (PT) and activated partial thromboplastin time (APTT). The percentage activity of FV (PFV) was lower than 3% in case 1. The mother of case 1 showed no tendency to bleed. She had mild prolongation of PT and APTT. The PFV was only 43%. Both cases were found to have the same novel mutation in F5, which was c.5419G>A (p.Ala1807Thr) in exon 16. The variant in case 1 was inherited from the mother of case 1. Whole-exome sequencing (WES) also found a splice site mutation: a 103 Mb maternal uniparental disomy (UPD) of 1q21.1-qter in case 1, in which the F5 gene is located in this segment. So case 1 was homozygote and the mother of case 1 was heterozygote. The novel mutation of F5 was predicted to be harmful by bioinformatics software including Sorting Intolerant From Tolerant (SIFT), Polyphen2, LRT, and Mutation Taster. In summary, c.5419G>A (p.Ala1807Thr) in exon 16 of F5 is a pathogenic mutation, which causes severe congenital FVD in homozygote patients.

## Introduction

Coagulation factor (F) V is a glycoprotein that is produced by hepatocytes and megakaryocytes, which is an essential component in the prothrombinase complex that activates the prothrombin to thrombin process (1, 2). FV is also known as a labile factor and protein cofactor. It was first described by Owren in Norway during World War II (3). FV protein consists of 2,224 amino acids (AAs) including a long signal peptide and a mature single-chain polypeptide (4, 5). Moreover, it is composed of six domains with molecular weight of 330 KDa, including A1, A2, B, A3, C1, and C2 (6). The gene encoding human FV is F5 (NCBI: MIM #612309), which is located on 1 at q21-25. It consists of 25 exons spanning about 80 kb, following transcription it gives rise to a 6.8 Kb mRNA. More than 150 different mutations including missense, nonsense, frameshift, and splice site mutations have been reported throughout the F5 gene (7). Congenital factor V deficiency (FVD), a rare autosomal recessive bleeding disorder, is attributed to F5 mutations. Bleeding is often mild in most cases, and life-threatening bleeding is rarely reported (8).

Uniparental disomy (UPD) refers to a condition in which both homologs of a chromosomal region/segment are inherited from only one parent. The incidence of UPD of any chromosome is estimated to be about 1:3,500 live births. UPD for some chromosomes can result in abnormality through aberrant genomic imprinting, defined as differential gene expression dependent on the parent of origin (9).

In this study, we summarized two cases with congenital FVD associated with a novel F5 pathogenic mutation and maternal UPD, and analyzed the relationship of the clinical features and genetic characteristics in congenital FVD.

## Methods

### Cases

Case 1 and the mother of case 1 with congenital FVD in a Chinese pedigree carried a novel F5 mutation. Their clinical manifestations, coagulation test, FV activity, and gene variations were analyzed.

### Variation Analysis

Written informed consent was obtained from the parents of the patient. This study was approved by the institutional review board of the Third Xiangya Hospital, Central South University. Overall, 5 mL of peripheral venous blood of the infant and parents was collected, and genomic DNA was extracted for whole-exome sequencing (WES) of family trios.

## Results

### Case Presentation

Case 1 was the proband. She was a 40 2/7 week gestation female infant, who was delivered by a 28-year-old gravida 2, para 1, aborta 1 woman *via* vaginal delivery. The infant was vigorous at birth with Apgar scores of 10 and 10 at 1 and 5 min, respectively, with a birthweight of 2,840 g (<10th percentile). Due to hematemesis, the patient was transferred to the neonatal intensive care unit (NICU) at 29 h after birth. Pregnancy was complicated by premature rupture of membranes 2 days before delivery. Maternal antenatal testing results were unremarkable. There was no familial history of coagulation disorder. When she was admitted to NICU, vital signs were within normal limits, and physical examination findings were unremarkable except ecchymosis at the injection sites and a large cephalohematoma. The blood routine test, urine routine test, stool routine test, liver function test, and renal function test were normal. She had significant prolongation of prothrombin time (PT) and activated partial thromboplastin time (APTT). The percentage activity of FV (PFV) was always lower than 3% (normal 81–160%; Table 1). Other coagulation/anticoagulation/fibrinolytic protein levels were normal. The abnormal coagulation function was temporarily corrected by transfusing fresh frozen plasma. Therefore, congenital FVD was diagnosed. The infant died of severe intracranial hemorrhage (ICH) at day 42 after birth.

**Table 1:** The clinical manifestations, coagulation test, and FV activity in the pedigree.

	**Bleeding**	**PT (9–14 s)**	**APTT (20–40 s)**	**Percentage activity of FV (%)**
Case 1 (proband)	ICH, ecchymosis	126 s	>150 s	<3
The mother of case 1	No	18 s	48 s	43
The father of case 1	No	11 s	25 s	98

*ICH, intracranial hemorrhage; PT, prothrombin time; APTT, activated partial thromboplastin time*.

The other case is the mother of case 1. She showed no tendency to bleed. However, she had slight prolongation of PT and APTT. The PFV was only 43% (Table 1).

### Molecular Genetic Analysis

**Figure 1:**
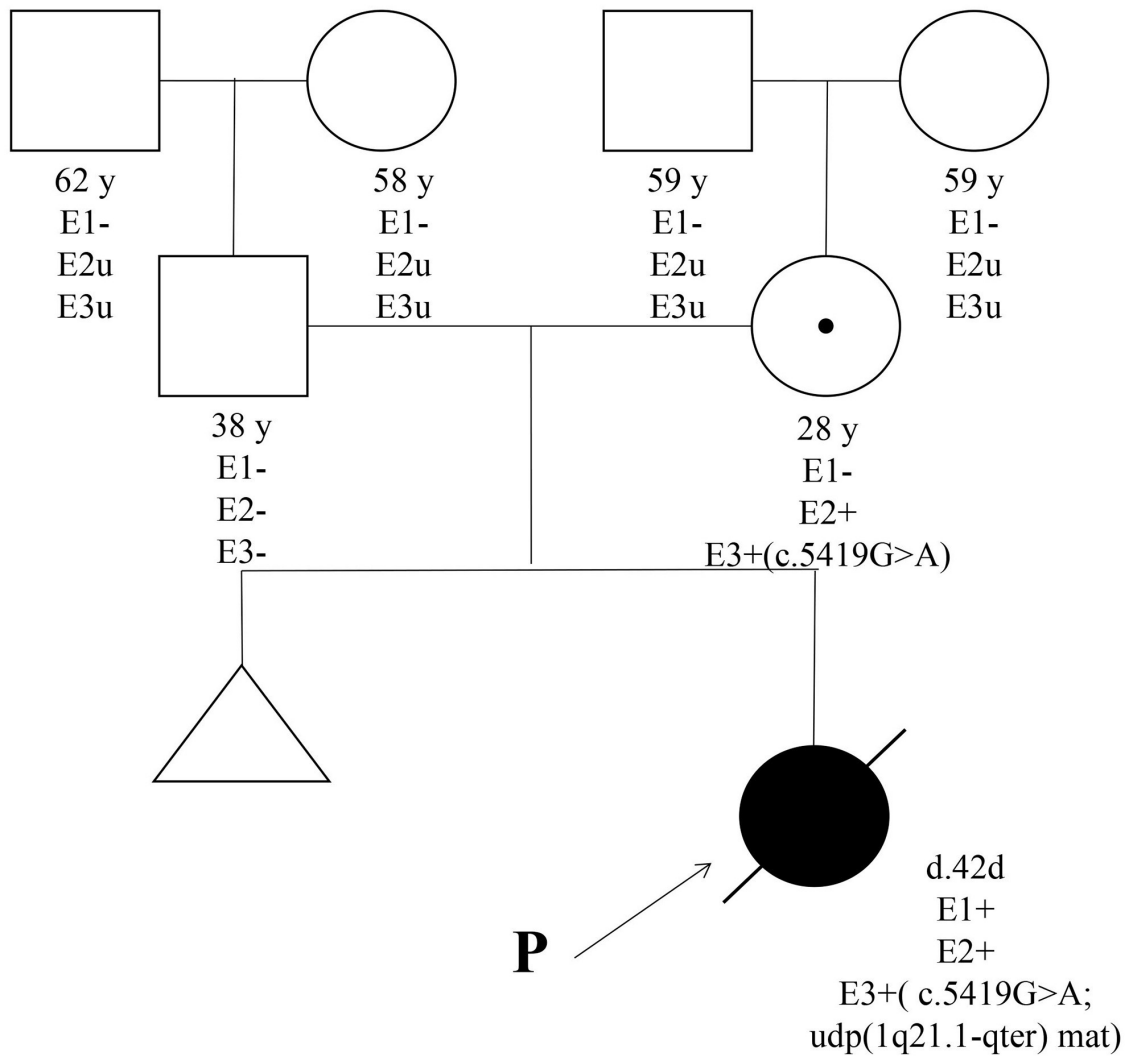
Pedigree. E1, physical examination; E2, coagulation test; E3, whole-exome next-generation sequencing; P, proband.

**Figure 2:**
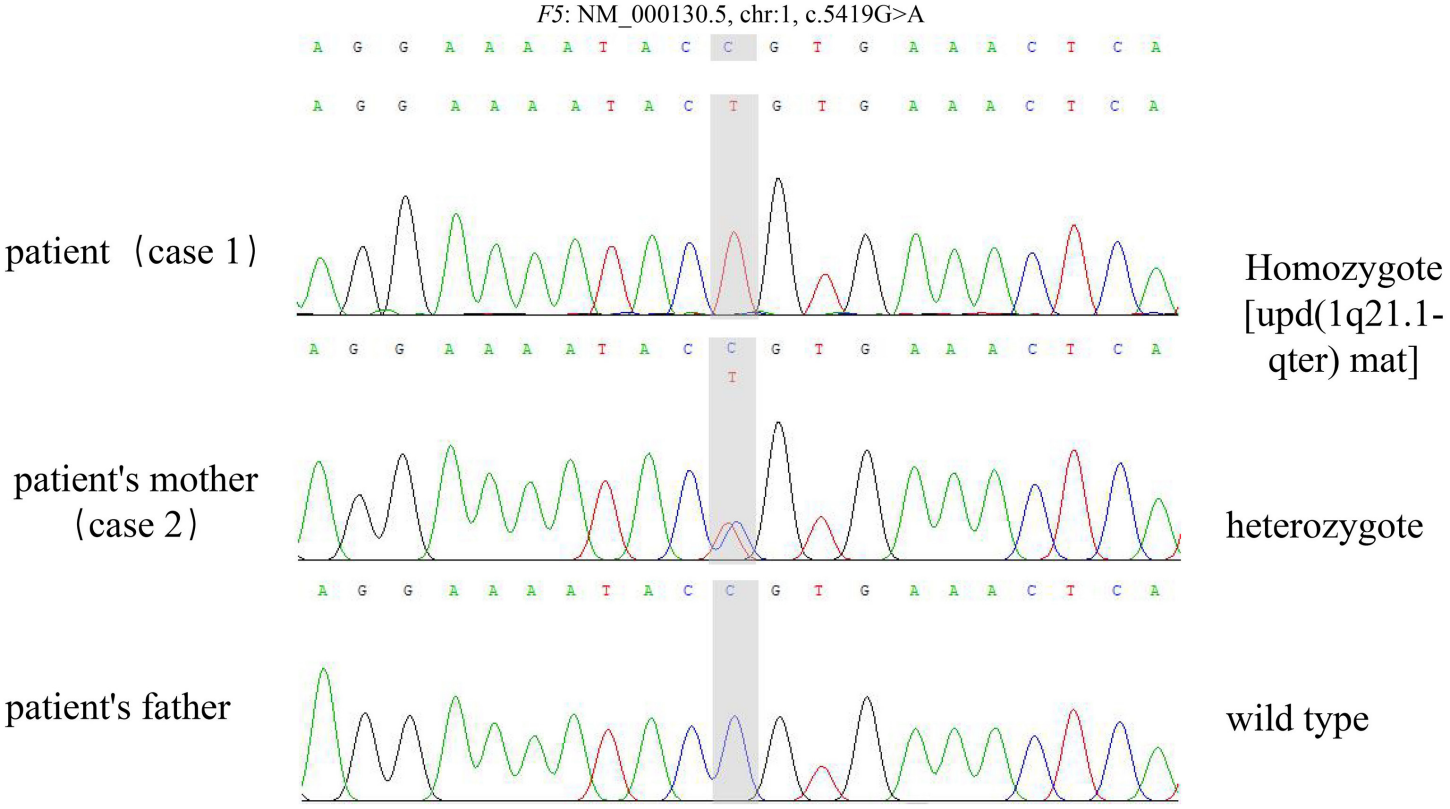
The sequencing of the trio.

Both cases were identified with the same novel mutation in F5, which was c.5419G>A (p.Ala1807Thr) in exon 16. The mutation in case 1 was inherited from the mother of case 1. WES also found maternal UPD of 1q21.1-qter [udp (1q21.1-qter) mat] in case 1, in which F5 is located. Therefore, case 1 was homozygote, and the mother of case 1 was heterozygote (Figures 1, 2).

p.Ala1807Thr, based on reference sequence NM_000130.4, is a splice site mutation. According to the guidelines of American College of Medical Genetic (ACMG), the Exome Aggregation Consortium (EXAC) database, and the Thousands Asian Genomes database, this genetic variation is not included. Furthermore, mutation pathogenicity prediction software, including SIFT, Polyphen2 (http://genetics.bwh.harvard.edu/pph2/), LRT, Mutation Taster (http://www.mutationtaster.org/), Predict SNP (https://loschmidt.chemi.muni.cz/predictsnp/), and FATHMM, predicted that the mutation of c.5419G>A is pathogenic or harmful, with a Rare Exome Variant Ensemble Learner (REVEL) value of 0.816.

**Figure 3:**
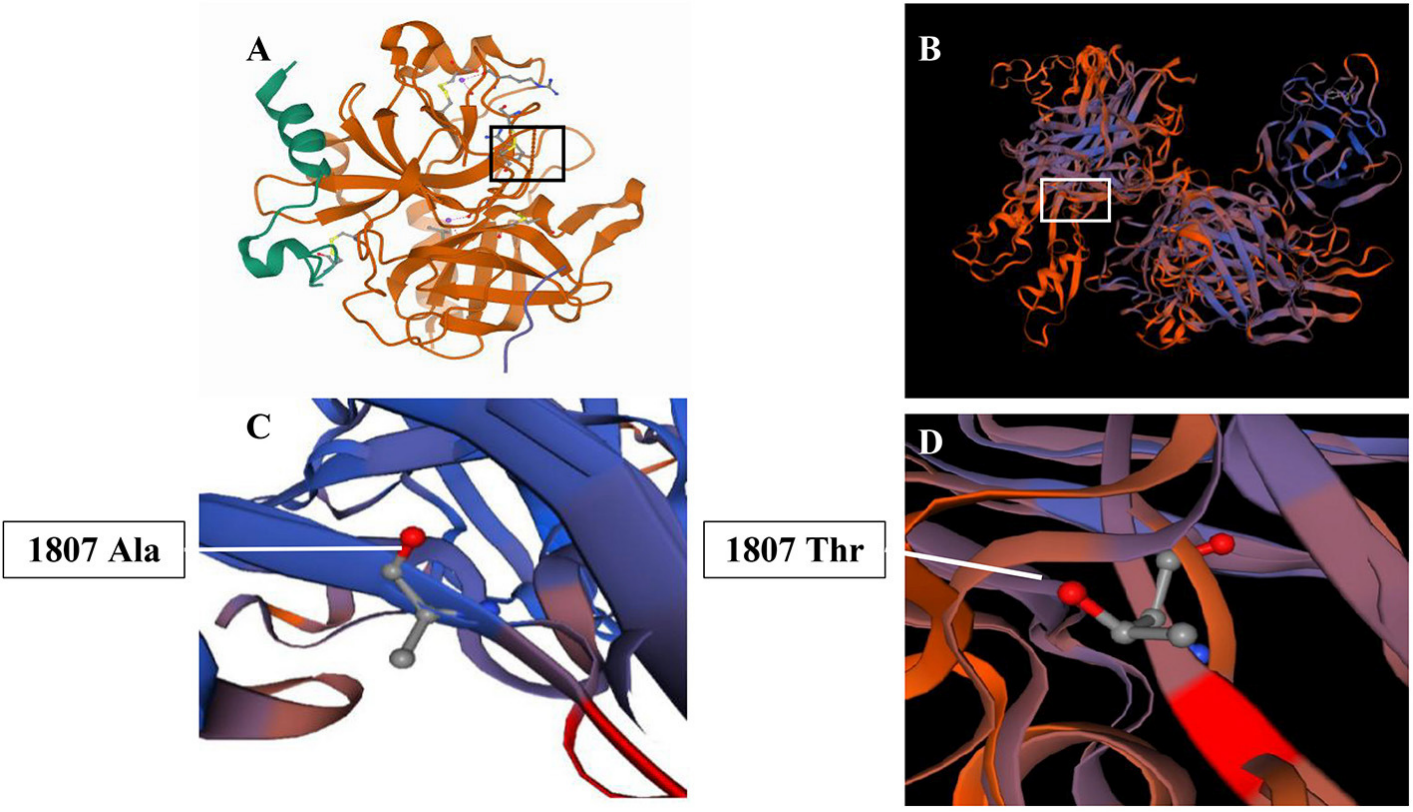
Three-dimension homology model changes induced by the c. 1351C>T mutation. **(A,C)** Wild-type mutation; **(B,D)** c. 1351C>T (p.Ala1807Thr) mutation.

The SWISS-MODEL workspace (http://swissmodel.expasy.org) was used to characterize the effect of the mutation on the protein of FV. Homology modeling of the amino acid was employed as the template of the Protein Data Bank (PDB) structure. We found that the structures of the protein resulting from the mutation of c.5419G>A were predicted and compared with that of the wild type, whereupon significant structural differences were observed (Figure 3). The predicted structure of the splice site mutation c.5419G>A (p.Ala1807Thr) in exon 16 revealed a significant abnormal structure of the protein that might cause protein dysfunction.

## Discussion

In hemorrhagic diseases overall, the prevalence of congenital FVD is 1:1,000 and 1:1,000,000 in heterozygote and homozygote patients, respectively. FVD from homozygous or compound heterozygous F5 mutations usually have FV levels lower than 10%, whereas those from a single heterozygous F5 mutation have FV levels typically around 50% (10, 11). Using the WES method, we identified that the mutation was located within a splice site mutation (c.5419G>A) in F5. The pathogenicity of this variant mutation was already determined in 1997, disturbing the correct splicing with a null allele (12). In this case, the homozygous condition of the patient might produce the absence of FV and justify the severe FV deficiency. Meanwhile, bioinformatics predicted that the mutation of c.5419G>A was harmful, and lead to the change of the spatial structure of the FV protein. Case 1 was homozygous, whose PFV <3%, and the mother of case 1 was heterozygous, whose PFV was 43%. Therefore, this splice site mutation should be a pathogenic mutation.

Congenital FVD is believed to be an autosomal recessive disorder. The splice site mutation in case 1 came from her mother alone. Due to maternal UPD, case 1 became homozygous and showed a severe clinical manifestation of congenital FVD. UPD includes two types: isodisomy and heterodisomy. Isodisomy describes the inheritance of two copies of a single parental homolog with associated reduction to homozygosity in the offspring, whereas heterodisomy refers to the inheritance of both homologs from one parent. In the case of isodisomy, homozygosity of autosomal recessively inherited mutations is possible.

In our cases, the severity of bleeding was related to the activity of FV. However, previous patients, whose activity of FV was very low, only had a mild bleeding tendency (13). In the case presenting in this report, this mutation in the homozygous state could cause a different severe phenotype: the fatal intracranial hemorrhage that the patient presented at day 42 after birth. However, as this patient had UPD of 1q21.1-qter, the patient could have had other genes affected. If these genes had mutations in the homozygous state, could these mutations also be involved in the clinical phenotype and cause severe intracranial hemorrhage? This requires further research. In addition, further investigation is needed to understand why this novel mutation seriously impaired FV protein function.

In summary, c.5419G>A of F5 is a pathogenic mutation, which causes severe congenital FVD in homozygote patients.

## Data Availability Statement

The datasets for this article are not publicly available due to concerns regarding participant/patient anonymity. Requests to access the datasets should be directed to the corresponding author.

## Ethics Statement

Written informed consent was obtained from the individual(s), and minor(s)' legal guardian/next of kin, for the publication of any potentially identifiable images or data included in this article.

## Author Contributions

LC developed the original idea, abstracted and analyzed the data, and wrote the manuscript. YL and WZ contributed to the conception of the work, the acquisition, analysis, and interpretation of the data for the work, and drafted the work. TB contributed to the conception and design of the work, and revised it critically for important intellectual content. All authors approved publication of the content and agreed to be accountable for all aspects of the work in ensuring that questions related to the accuracy and integrity of any part of the work are appropriately investigated and resolved.

## Conflict of Interest

The authors declare that the research was conducted in the absence of any commercial or financial relationships that could be construed as a potential conflict of interest.

## Publisher's Note

All claims expressed in this article are solely those of the authors and do not necessarily represent those of their affiliated organizations, or those of the publisher, the editors and the reviewers. Any product that may be evaluated in this article, or claim that may be made by its manufacturer, is not guaranteed or endorsed by the publisher.
