# Supplementary material for: Case Report: Novel Mutation of F5 With Maternal Uniparental Disomy Causes Severe Congenital Factor V Deficiency
Source: Front Pediatr. 2022 Jun 7;10:913050. doi: 10.3389/fped.2022.913050 (PMC9211043; doi:10.3389/fped.2022.913050)

Supplementary Material

1 Supplementary Data

We showed some detail about the data analysis pipeline used for exome analysis.

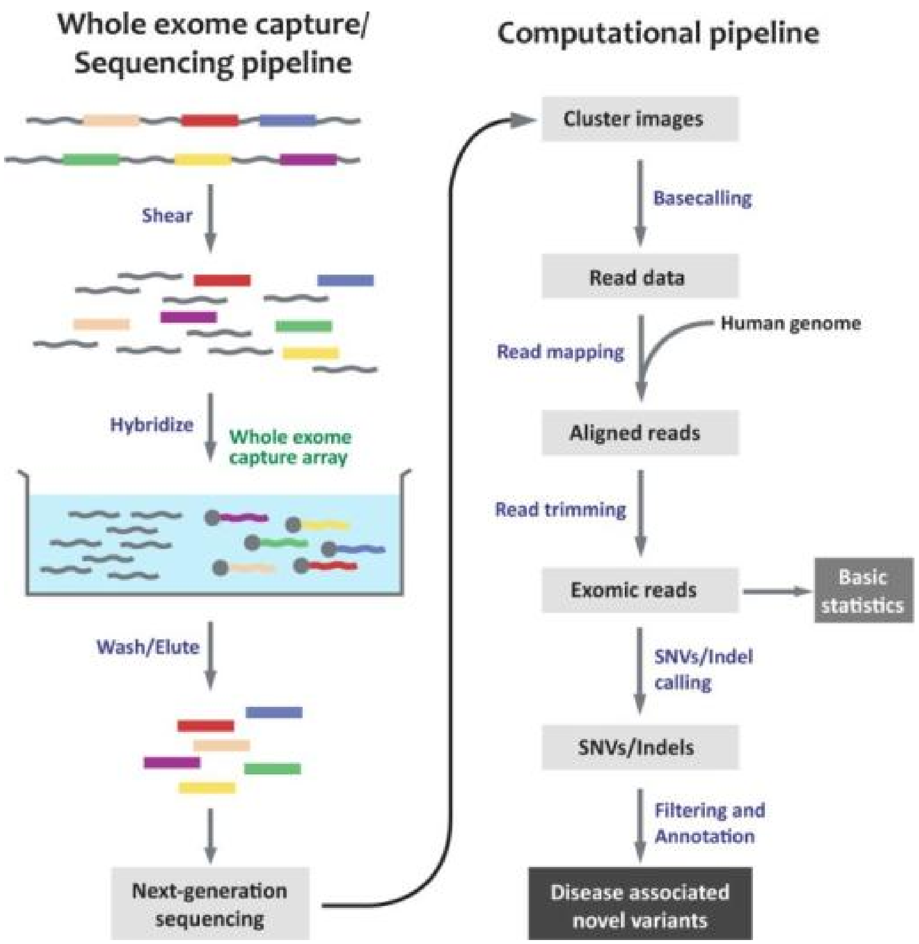

Supplement: Supplementary file 1 [file Data_Sheet_1.PDF]
